# Factors influencing pharmacists' participation in continuing education activities in the United Arab Emirates: insights and implications from a cross-sectional study

**DOI:** 10.1186/s40545-023-00623-3

**Published:** 2023-10-02

**Authors:** Khalid Awad Al-Kubaisi, Asim Ahmed Elnour, Adel Sadeq

**Affiliations:** 1 Department of Pharmacy Practice and Pharmacotherapeutics, College of Pharmacy, University of Sharjah, P. O. Box 27272, Sharjah, United Arab Emirates; 2 Clinical Pharmacy Program, College of Pharmacy, Al Ain University (AAU), Abu Dhabi Campus, Abu Dhabi, United Arab Emirates; 3 AAU Health and Biomedical Research Centre, Al Ain University, Abu Dhabi, United Arab Emirates; 4 Program of Clinical Pharmacy, College of Pharmacy, Al Ain University, Al Ain, United Arab Emirates

**Keywords:** Continuing education, Pharmacists, Perception, Predictors, United Arab Emirates

## Abstract

**Background:**

Continuing professional development (CPD) is essential for pharmacists to maintain and enhance their knowledge and skills. The purpose of this research was to collect data about the perception of pharmacists in the United Arab Emirates (UAE) towards CPD and identify factors that motivate or hinder their participation in different types of CPD activities.

**Methods:**

A cross-sectional survey was conducted among 322 pharmacists who completed a self-administered questionnaire that assessed their demographic characteristics, CPD preferences, motivators and obstacles to attending CPD programs, and perceived learning outcomes.

**Results:**

Participants’ average age was 33 years (mean = 30.6, SD = 5.97), and the range of years, since they graduated from a pharmacy degree program was 18 years (mean = 10.9, SD = 4.8). More than half of the participants were female; 198 (61.5%) and 193 (59.9%) of them were married. The study found that married pharmacists (AOR = 0.5, 95% CI 0.266–0.939, *P* value = 0.031), older participants (AOR = 0.232, 95% CI 0.266–0.939, *P* value = 0.04), and those who graduated longer than 16 years ago were less likely to attend live CPD events (AOR = 0.454, 95% CI 0.22–0.924). However, participants who worked up to 15 h had higher odds of attending live CPD events (AOR = 3.511, 95% CI 1.117–11.039, *P* value: 0.026). In addition, female pharmacists were less likely to participate in computer/internet-based continuing education than male pharmacists (AOR = 0.038, 95% CI 0.293–0.965, *P* value = 0.038). It also revealed that pharmacists who were not motivated by the topic of the CPD activity had a higher chance of attending computer/internet-based format (AOR = 2.289, 95% CI 1.198–4.371, *P* value = 0.012). In contrast, those who did not report the long distance to the CPD site as a hindrance had a lower likelihood of attending online internet-based CPD (AOR = 0.548, 95% CI 0.319–0.941, *P* value = 0.029).

**Conclusions:**

This study is the first to explore pharmacists’ predictors of attending different CPD activities. These predictors are gender, age, marital status, experience since graduation, working hours, family barriers, work responsibilities, interest in the presentation topic, and the long travel distance to the site. These findings suggest that pharmacists have unique challenges and motivations regarding continuing education and that tailored approaches may be necessary to encourage participation.

## Background

Continuing professional development (CPD) is essential for pharmacists to stay up-to-date with new medications, technologies, and regulations [1–3]. It also helps them enhance their clinical skills, communication skills, and patient counseling abilities [4, 5]. In addition, as healthcare systems evolve and technology advances, pharmacists must be proficient in using electronic health records and other digital tools to provide safe and effective medication management [5–11]. Furthermore, state licensing boards and professional organizations often require CPD [12–14]. For example, pharmacists may be required to attend CPD courses or complete certification programs to stay updated on new drugs, treatments, and regulations [12, 15, 16]. Therefore, continuing education is crucial for the pharmacist's professional growth and safety and for providing patients with the highest level of care [5, 17].

CPD can be defined as an “educational activity designed to increase and maintain the competence of pharmacists” [18]. It can occur through in-person or online activities, such as webinars [1, 19–21]. Effective CPD programs must consider pharmacists' requirements and preferences [22]. When pharmacists are provided with topics that interest them, they are more likely to remember the information if they actively engage with it, such as by taking notes or participating in discussions [23, 24]. Therefore, many studies focused on understanding pharmacists' views, perceptions, and practices towards attending CPD activities in the Gulf region and Middle Eastern countries [23, 25–30]. In Kuwait, for example, a survey showed that more than half of the pharmacists (60%; *n* = 246) have good to excellent attitudes towards CPD and that seminars were the most preferred activity [23]. In addition, another study explained that just under three quarters (71%; *n* = 207) of pharmacists in Qatar had not participated in any CPD activities on fetal medication usage [30]. On the other hand, previous studies in Saudi Arabia [31, 32], Lebanon [27, 28, 33–35] reported that lack of time, occupational restrictions, and expense were the main obstacles to CPD participation.

There is a growing demand for CPD programs for pharmacists in the UAE. For instance, the Dubai Health Authority (DHA) offers a variety of options to meet the demand for CPD among pharmacists. These options include both open live activities and self-study activities [36]. Open live activities such as courses, seminars, symposia, meetings, and conferences provide pharmacists with interactive learning experiences. On the other hand, self-study activities such as accredited distance e-learning programs allow pharmacists to learn at their own pace and convenience [37]. More importantly, pharmacists in the UAE must complete a certain number of hours of CPD as mandated by the Ministry of Health and Prevention (MOHAP). In-charge and second pharmacists must complete 20 h, while assistant pharmacists must complete 10 h [38]. These requirements ensure that pharmacists stay updated and maintain their professional competence. Compliance is necessary to preserve pharmacists’ licenses and continue practicing in the UAE. The DHA and MOHAP in the UAE recognize various local, regional, and international accrediting bodies. Locally, they acknowledge bodies, such as the MOHAP, Health Authority-Abu Dhabi (HAAD), and the University of Sharjah. Regionally, they recognize bodies, such as the Saudi Commission for Health Specialties and the Kuwait Institute for Medical Specialization. Internationally, they acknowledge accrediting bodies in Asia, the USA, the UK, and Canada (e.g., the Singapore Medical Council and the American Council on Pharmaceutical Education) [36].

Despite the substantial evidence available from previous studies conducted in various countries [23, 25–30, 35, 39–44], there is a notable scarcity of research investigating the predictors that impact their participation in these programs, specifically within the UAE. This research gap represents an opportunity to generate empirical evidence specific to the UAE context, shedding light on the unique barriers, motivators, and preferences influencing pharmacist participation in CPD programs. Therefore, the purpose of this research was to collect data about the perception of pharmacists in the UAE towards CPD, factors associated with attending CPD activities, and predictors that motivate/hinder pharmacists’ participation in four types of CPD activities (live, online, printed materials and video/audio formats presentations). By identifying these factors, policymakers, educational institutions, and professional bodies can design targeted strategies to promote and enhance pharmacist engagement in CPD activities in the UAE and the region and ultimately improve the quality of pharmaceutical care provided to patients.

## Methods

### Study design and population

This cross-sectional study was undertaken between July and August 2022. A random sample of 372 pharmacists out of 11,153 registered in the UAE in 2019 [38]. This sample was based on a 95% confidence level and a confidence interval of 5%. Pharmacists with at least 1 year of professional work experience were included in this study. In contrast, those with less than 1 year of skilled work experience, administrative staff, pharmacy students, and other healthcare professionals were excluded as they were beyond the scope of this study.

The sample was randomly selected from the address list of all registered community pharmacies in the Yellow Pages directory by entering the names of pharmacies in the four emirates of the UAE (Abu Dhabi, Dubai, Sharjah, and Ajman) into an Excel spreadsheet and assigning each one a number. From there, a random number generator was used to select the sample size of 351 pharmacies. This method ensured that the sample was representative of the entire population of pharmacies in the UAE and minimized any potential bias in the selection process. The next step was to contact each of the selected pharmacies and request their participation in the study.

The researchers deliberately chose to personally distribute surveys to community pharmacies in the UAE based on a strategic decision, considering the unreliability of postal services. This approach allowed for direct communication and ensured a higher response rate. Three 4-year pharmacy students from the University of Sharjah, faculty of pharmacy-UAE, were recruited to collect the data. They were females and spoke both Arabic and English languages. They provided three training sessions, each lasting around 45 min, about the university’s ethical guidelines and the principles for conducting surveys. The training involved meeting the students in 3 days to explain the scope of the study and their role and clarify the use of the assessment framework. The training also focused on teaching active communication and listening skills while avoiding unintentional leading or suggestive behavior to ensure accurate data collection.

The student researchers approached the pharmacists during their working hours in the pharmacy. They introduced themselves as independent student researchers separate from the MOH, DHA, and other health authorities. The student researchers provided the pharmacists with an explanation of the purpose of the study and its objectives. Participants were assured voluntary participation, anonymity, confidentiality, and no career impact, ensuring comfort and willingness to participate in the study. The participants were also made aware that no correct or incorrect answers existed. Overall, the survey participation process involved meeting the inclusion criteria and agreeing to participate. If they agreed to participate, one pharmacist from each pharmacy was asked to fill out the questionnaire independently, with the option to seek assistance if necessary. When a pharmacy declined participation, the student researchers sought the nearest alternative pharmacy.

### Data collection-questionnaire

#### Research instruments

The questionnaire was adapted from a previously published validated tool [35, 44]. It was in English, created in Google Forms, and comprised 19-questions divided into seven sections. The first section of the questionnaire consists of questions regarding sociodemographic characteristics. For example, the gender, age, marital status, educational qualification of the pharmacists, area of practice (chain pharmacy, independent pharmacy, and hospital pharmacy), working hours, and country of course. This section of the survey also asked if the pharmacist is a member of any professional organizations, if CPD is required to renew the license, and if the employer places a high value on their involvement in CPD.

The second section focused on the preferred type of CPD activities in the previous 6 months, including in-person attendance, online attendance, interactive workshops, printed educational materials, Audio/video-recorded formats presentations, and effective advertising. Each activity scored 1 (yes) or 0 (no). The third section concerned the satisfaction levels of the pharmacists with the preferred type of CPD activities in the previous 6 months, which were coded as an ordinal variable: very highly satisfied = 1, highly satisfied = 2; satisfied = 3; less satisfied = 4, and not satisfied = 5.

The fourth section focused on the motivators of attending CPD programs, which included interest in the topic of the presentation, low or no cost/fees of registration, opportunities for networking and socializing, effective advertising, and the CPD offered during a conference [20]. Each motivator was answered with a yes (1 response) or no (0 replies). The fifth section discussed why pharmacists might not attend the CPD program, such as work and family commitments, lack of time, lack of interest in the topic, lack of financial resources, inaccessibility of free CPD, and the convenience of receiving print and electronic materials. Responses were coded either 1 (yes) or 0 (no) for each obstacle.

Topics in CPD accounted for the sixth section were adopted and developed from previously published questionnaires [19, 20]. They included new disease management approaches, pharmacy practice innovations, humanities or psychology topics, longitudinal programming, and certification and skill development. The responses were graded using a Likert scale that ranged from 1 to 5, with 1 representing low interest and 5 representing high interest. Finally, the seventh section asked which organization was responsible for the CPD programs' content and quality, such as health authorities, colleges/faculties, local providers/sponsors, and pharmacy employers, was adopted from a previously published questionnaire [39]. Responses on each criterion were either 1 (yes) or 0 (no) for each item.

The questionnaire underwent a rigorous review and modification to ensure face and content validity. This included assessments by two co-authors and faculty members in the field of CPD and piloting with six pharmacists to provide clarity and readability. The modifications made to the questionnaire focused on language to ensure that it was clear and understandable. The revised response options for measuring participants' interests were changed to provide more explicit and precise descriptors. The original options were replaced with "low interested," "somewhat uninterested," "neutral," "somewhat interested," and "highly interested." This modification aimed to address concerns about potential ambiguity in the original response options and allow participants to express their level of interest more accurately. The question of the current practice in the published initial questionnaire of five responses compromised (community pharmacy, hospital, academic, industry or research center, marketing/sales or drug stores, and other (e.g., regulatory affairs, insurance companies) were modified into two responses: a chain pharmacy and an independent pharmacy. However, based on the concerns raised by two pilot study participants, it was decided to add a question to investigate whether community pharmacists have had previous experience in hospitals. This was done to ensure that their responses were not influenced by their hospital experience, as evidence-based knowledge is essential to the job description in hospital pharmacies. This addition was made to gather more accurate and unbiased participant data.

### Ethical approval

The University of Sharjah, UAE, approved this study (REC-22-04-07-02, 2nd June 2022). Before participating, each participant gave their informed consent.

### Data analysis

The data from the study were analyzed using SPSS version 26. Descriptive statistics were used to analyze categorical and continuous variables, while the Chi-square test and binary logistic regression were conducted to identify predictors of attending CPD activities. A significance level of *P* < 0.05 was used to determine statistical significance.

## Results

322 of 372 pharmacies (86.5%) agreed to participate. The average age of the participants was 33 years (mean 30.6, SD = 5.97), and the range of years, since they graduated from a pharmacy degree program was 18 years (mean 10.9, SD = 4.8). More than half of the participants (*n* = 198 (61.5%) were female, and 193 (59.9%) were married. A hundred twenty-two (62.7%) were working in chain pharmacies, approximately one in three (29.2%) in independent pharmacies, and almost one in ten (8.1%) were from hospital pharmacies. Most participants (*n* = 265, 82.3%) have a bachelor's degree as their highest degree, and 312 (96.9%) had their pharmacy practice in the UAE. 84% of the participants worked more than 30 h weekly, and 38.2% were professional organization members. Four out of five participants (80.4%) stated that their employers valued their participation in the CPD activities. Demographic characteristics are summarized in Table 1.

**Table 1:** Demographic distribution characteristics of the participants (*N* = 322)

Characteristic	Frequency (%)
*Age (years)*	
20–30	212 (65.8)
31–40	84 (26.1)
> 40	26 (8.1)
*Gender*	
Female	198 (61.5)
Male	124 (38.5)
*Marital status*	
Single	129 (40.1)
Married	193 (59.9)
*Education level*	
B.Pharm	265 (82.3)
Diploma	2 (0.6)
Pharm D	21 (6.5)
Master	34 (10.5)
*Area of practice*	
Chain pharmacy	202 (62.7)
Independent pharmacy	94 (29.2)
Hospital pharmacy	22(8.1%)
*Working hours/week*	
< 15	0 (0)
15–30	50 (15.5)
> 30	272 (84.4)
*Previous country of practice*	
UAE	312 (96.9)
Syria	1 (0.3)
India	6 (1.9)
Philippines	1 (0.3)
Egypt	2 (0.6)
*Are you a member of a professional organization?*	
Yes	123 (38.2)
No	199 (61.8)
*Does the Employer value your CPD participation?*	
Yes	259 (80.4)
No	63 (19.6)

### Types of CPD programs

**Fig. 1:**
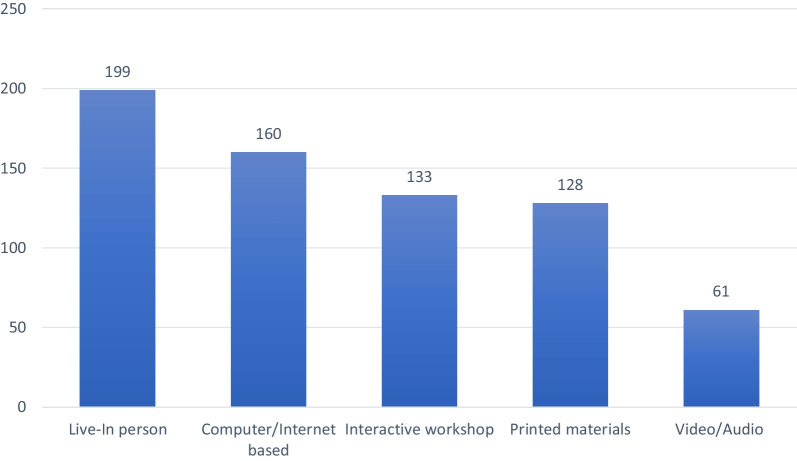
Types of CPD programs (*n* = 322)

Participants were surveyed about the types of CPD activity they had used within the past 6 months or were interested in using in the future (Fig. 1). More than half (*n* = 199, 61.8%) chose live-in-person attendance, followed by 160 (49.7%) who decided online attendance. Furthermore, the interactive workshop was also a popular choice (*n* = 128, 41.3%), while reading printed materials (*n* = 128, 39.8%) was surprisingly reported more frequently than watching Video/Audio formats 61 (*n* = 61, 18.9%).

### Motivators and barriers to attending CPD programs

**Fig. 2:**
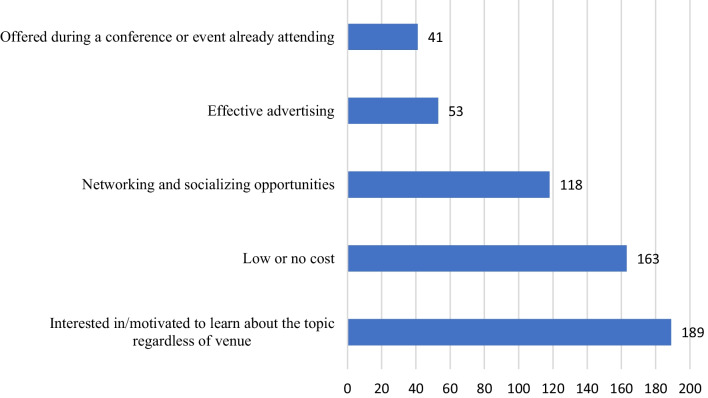
Motivation for attending CPD (*n* = 322)

The study highlights some motivators for attending CPD. First, the topic (*n* = 189, 58.7%) and the free cost of CPD (*n* = 163, 50.6%), were the primary motivators for participants to participate. In addition, around one-third of the participants (36.6%) reported that attending CPD activities provided them with networking and socializing opportunities (Fig. 2). However, participants identified work responsibilities (*n* = 181, 56.2%) and family commitments (*n* = 153, 47.5%) as the biggest obstacle to participating in CPD activities. The high cost of CPD activities (*n* = 127, 39.4%) and the distance required to travel to the site (*n* = 115, 35.7%) were also cited as significant barriers. Barriers to joining CPD activity are displayed in Table 2.

**Table 2:** Barriers to attending CPD (*N* = 322)

Barriers	Frequency (%)
Work responsibilities	181 (56.2)
Family commitments	153 (47.5)
Cost	127 (39.4)
Distance to travel	115 (35.7)
Timing of talk	94 (29.2)
Availability of free CE	83 (25.8)

### Interest

The majority of pharmacists (*n* = 290, 96.7%) were eager to attend (CPD) activities that offer certificate programs and cover innovations in pharmacy practice (*n* = 288, 96.0%). They also want to join CPD activities related to disease management (*n* = 286, 95.3%) and humanities or psychology topics (286, 95.3%). Table 3 summarizes pharmacists’ interests.

**Table 3:** Interests of pharmacists towards CPD topics (*N* = 322)

Interests	Frequency (%)
*Results in certification or skill development*	
Low interested	10 (3.3)
Somewhat uninterested	40 (13.3)
Neutral	60 (20)
Somewhat interested	80 (26.7)
Highly interested	110 (36.7)
Total	290(96.7)
*Covers innovations in pharmacy practice*	
Low interested	3 (1.0)
Somewhat uninterested	12 (4.0)
Neutral	24 (8.0)
Somewhat interested	48 (16.0)
Highly interested	201 (66.6)
Total	288 (96.0)
*Covers innovations in disease management*	
Low interested	5 (1.7)
Somewhat uninterested	11 (3.8)
Neutral	22 (7.7)
Somewhat interested	45 (15.7)
Highly interested	203 (71.0)
Total	286 (95.3)
*Covers humanities or psychology topics*	
Low interested	3 (1.0)
Somewhat uninterested	11 (3.9)
Neutral	22 (7.7)
Somewhat interested	45 (15.7)
Highly interested	205 (71.7)
Total	286 (95.3)
*Covers longitudinal program o low interested*	
Low interested	4 (1.5)
Somewhat uninterested	12 (4.5)
Neutral	23 (8.6)
Somewhat interested	54 (20.1)
Highly interested	175 (65.3)
Total	268 (89.3)

### Quality and content of CPD programs

Most pharmacists (*n* = 281, 87.3%) believe that health authorities are primary providers of high-quality CPD programs. However, approximately two-fifths of them (*n* = 118, 36.6%) agree that colleges/faculties also play an integral part in maintaining the topics covered in CPD programs and emphasizing both their quality and relevance, as indicated in Table 4.

**Table 4:** Responsible organizations for the quality and content of CPD programs (*n* = 322)

Responsible organization	Frequency (%)
Health authority	281 (87.3%)
Colleges/faculties	118 (36.6%)
Local providers/sponsors	81 (25.2%)
Employers of pharmacy	68 (21.1%)

### Bivariate analysis of factors associated with attending CPD activities

The Chi-square test for association was conducted between socio-demographic characteristics and the four types of CPD activities (Table 5). First, we observed an association between attending live-in-person CPD activities and the age group of pharmacists (*P* value = 0.056), years of pharmacists’ graduation (*P* value = 0.000), and area of pharmacists’ practice (*P* value = 0.054). Second, attending an online-internet-based CPD was significantly associated with the location of the course (*P* value = 0.027) and the pharmacists’ employer value of CPD (*P *value = 0.040). Third, there was a statistically significant association between attending workshops and CPD activities and the gender of the pharmacist (*P* value = 0.058) and belonging to a professional organization (*P* value = 0.024). Finally, we did not observe any associations between socio-demographic characteristics and watching DVD/video/audio CPD activities or reading printed materials.

**Table 5:** Bivariate analysis of factors associated with attending CPD activities (*n* = 322)

CPD activities	Variable	Yes (*n*/%)	No(*n*/%)	*P* value	*df*	*χ*^2^
	*Age group*					
	20–30	131(61.8%)	81(38.2%)	0.056	2	7.554
	31–40	54(64.3%)	30(35.7%)			
	> 40	14(53.8%)	12(46.2%)			
Live-in-person activities	*Graduation years*					
	1–5 years	24(53.3%)	21(46.7%)	0.000	2	18.213
	6–10 years	75(56%)	59(44%)			
	11–15yeras	58(68.2%)	27(31.8%)			
	16–20 years	42(72.4%)	16(27.6%)			
	*Area of practice*					
	Chain pharmacy	121(60.8%)	81(65.9%)	0.054	2	5.823
	Independent pharmacy	66(33.2%)	28(22.8%)			
	Hospital pharmacy	12(6%)	14(11.4%)			
Online-computer/Internet-based CPD	*Area of practice*					
	Chain pharmacy	112(70%)	90 (55.6%)	0.027	2	7.255
	Independent pharmacy	37(23.1%)	57(35.2%)			
	Hospital pharmacy	11(6.9%)	15(9.3%)			
	*Employer value CPD*					
	Yes	24(15%)	39 (24.1%)	0.040	1	4.212
	No	136(85%)	123 (75.9%)			
Workshop CPD	*Gender*					
	Male	17 (27.9%)	107(41%)	0.058	1	3.598
	Female	44(72.1%)	154(59%)			
	*Professional organization*					
	Yes	30 (49.2%)	169(64.8%)	0.024	1	5.078
	No	31(50.8%)	92(35.2%)			

### Key factors influencing attendance at CPD Activities

#### Factors affecting attendance at live in-person CPD activities

The study identified six predictors for attending live-in-person CPD. First, married pharmacists were less likely to participate in live CPD than single participants (AOR = 0.5, 95% CI 0.266–0.939, *P* value = 0.031). Furthermore, participants between the age of 31–40 and those above 40 years had lower odds of attending live CPD compared to younger participants at the age of 20–30 years (AOR = 0.232, 95% CI 0.266–0.939, *P* value = 0.04; AOR = 0.388, 95% CI 0.182–0.829, *P* value = 0.015). On the other hand, participants who had graduated longer than 16 years prior were less likely to attend live CPD compared to newly graduated pharmacists (AOR = 0.454, 95% CI 0.22–0.924, *P* value = 0.029). In addition, participants who work up to 15 h had higher odds of attending live-in person CPD than those with longer hours (AOR = 3.511, 95% CI 1.117–11.039, *P *value: 0.026). The results of the multivariable analysis are shown in Table 6.

**Table 6:** Multivariate models for key factors of attending CPD activities (*N* = 322)

CPD activities	Variable		*AOR*	95% *CI*		*P *value
				Lower bound	Upper bound	
Live-in-person	Age group2 (ref—20–30 years)	Age group (31–40)	0.232	0.058	0.934	0.04*
	Age group3 (ref—20–30 years)	Age group above 40 years	0.388	0.182	0.829	0.015*
	Marital status (ref—single)	Married	0.500	0.266	0.939	0.031*
	Graduation year (ref—6–10 years)	Graduation year (16–20)	0.454	0.223	0.924	0.029*
	Working hours (ref—. > 40 h)	Working hours up to 15 h	3.511	1.117	11.039	0.032*
	Motivation (ref—topic)	Do not motivate by the topic	0.524	0.297	0.926	0.026*
Workshop	Barrier (ref—have family responsibility)	Do not have a family barrier	2.289	1.198	4.371	0.012*
	Barrier (ref—have work responsibility)	Do not have a work responsibility barrier	3.477	1.195	10.112	0.022*
	Motivation (ref—topic)	Do not motivate by the topic	0.557	0.325	0.955	0.033*
Online-Internet-based	Gender (ref—male)	Female	0.038	0.293	0.965	0.038*
	Motivation (ref—motivated by the topic)	Do not motivate by the topic	2.289	1.198	4.371	0.012*
	Barrier (ref—have distance)	Do not have travel distance barriers	0.548	0.319	0.941	0.029*

^*^Statistically significant *P *value

#### Factors associated with attending workshops CPD activities

The study also identified three predictors for attending workshops (Table 6). Pharmacists who did not experience family-related barriers had higher odds of attending the seminar than those who did face family-related obstacles (AOR = 2.289, 95% CI 1.198–4.371, *P* value = 0.012). Similarly, pharmacists who did not have additional work responsibilities such as managerial or administrative duties or participation in committees or professional organizations were more likely to attend workshops CPD than those who did (AOR = 3.477, 95% CI 1.195–10.115, *P* value = 0.022). In addition, pharmacists who were not motivated by the topic had lower odds of participating in the workshop than those who did not (AOR = 0.557, 95% CI 0.325–0.955, *P* value = 0.033).

#### Factors associated with attending online computer/internet-based CPD activities

Female pharmacists were less likely to attend computer/internet-based CPD compared to male pharmacists, with a statistically significant adjusted odds ratio (AOR) of 0.038 (95% confidence interval [CI] 0.293–0.965, *P* value = 0.038). Furthermore, pharmacists who were not motivated by the topic of the CPD activity had a higher chance of attending computer/internet-based format, with an AOR of 2.289 (95% CI 1.198–4.371, *P* value = 0.012), compared to those who were motivated. In addition, pharmacists who did not report the long distance to the CPD site as a hindrance had a lower likelihood of attending online internet-based CPD, with an AOR of 0.548 (95% CI 0.319–0.941, *P* value = 0.029), compared to those who reported hindrance as displayed in Table 6.

## Discussion

Our study is the first to examine the determinants of pharmacists participating in four types of CPD activities. Importantly, we identified nine predictors for attending different types of CPD programs. These predictors are gender, age, marital status, experience since graduation, working hours, family barriers, work responsibilities, interest in the presentation topic, and the long travel distance to the site. Our results offer valuable insights into the factors influencing pharmacists' decisions to participate in CPD activities, ultimately leading to more educated and engaged pharmacists.

The present study found that live on-site continuing education activity CPD activity attendance was preferred over virtual/online attendance. This finding supports the result obtained by another study conducted in the United States before the COVID-19 pandemic [45]. More than half of pharmacy students and pharmacists (*n* = 149) surveyed in the United States believe that traditional face-to-face learning environments are more effective in knowledge acquisition than online settings [45]. The study also suggested that online learning may not always provide a satisfactory experience for students, emphasizing the need for further improvement and adaptation in the online education system. One explanation is that in a face-to-face setting, participants can ask questions, receive immediate feedback, and engage in discussions with their peers and instructors, which may enhance their understanding and retention of the material [46]. In addition, face-to-face learning may allow participants to network and build relationships with their peers and instructors, which may be particularly important in healthcare professions, where collaboration and teamwork are essential [47]. The study's findings emphasize that interactive and social learning experiences are particularly beneficial in fields, where collaboration and teamwork are essential. The health authorities can consider incorporating activities that foster networking, such as group projects, workshops, or professional communities of practice. These initiatives can facilitate knowledge sharing, the exchange of best practices, and the development of professional relationships, ultimately contributing to the growth and advancement of the pharmacy profession in the UAE.

We found that the primary motivator for participants to attend was the topic of CPD. These results match previous research on factors that motivate pharmacists’ participation in CPD [36, 37, 48, 49]. For example, a study in Lebanon found that most pharmacists (*n* = 107, 80.6%) participated in CPD based on their interest in the topic [50]. However, these barriers can be overcome by incorporating more engaging and relevant topics into continuing education programs. In addition, including interactive elements in these programs, such as quizzes, case studies, and simulations, can help to make the content more engaging and improve retention. This study also suggests that workshop organizers should consider these factors to maximize attendance and engagement when planning future events.

However, our study also found that work responsibilities and family commitments were the biggest obstacles to participating in CPD activities. Furthermore, we observed pharmacists without family barriers and work responsibilities were likelier to attend workshops. In addition, the high cost of CPD activities and the distance required to travel to the site were also cited as significant barriers. For example, pharmacists with travel distance barriers were more likely to attend online-virtual CPD. These results match previous research on factors that hinder pharmacists’ participation in CPD [35, 37, 42, 50]. For instance, a survey by Iskandar et al. found that the most cited barriers to attending CPD programs by Lebanese pharmacists were mainly work responsibilities (76%), travel distance (65.6%), and family commitments (48.4%) [50]. Similarly, research conducted in Lebanon demonstrated that family and work obligations were the most prevalent obstacles to attending CPD activities [51]. One explanation of this finding is that online internet-based CPD offers the convenience of completing courses at the pharmacist's pace and schedule. This flexibility allows pharmacists to balance their work and personal commitments while fulfilling their continuing education requirements. These findings highlight the importance of addressing the barriers that prevent pharmacists from accessing CPD opportunities, especially for those with family and work responsibilities. By doing so, pharmacists can maintain their knowledge and skills, benefiting patient care and outcomes.

Our results also suggest that age plays a significant role in choosing the CPD format. More specifically, we found that older pharmacists with more experience were less likely to attend live on-site CPD for pharmacists. Furthermore, the results also suggest that participation increases with age. This finding is consistent with Glazier et al. [52], who found that older participants were significantly more inclined towards online courses than younger participants in the United States. In addition, our findings support the results from Lebanon [51], indicating that age is not a constraining factor for pharmacists' participation in the CPD system. This is a positive sign for the pharmacy, demonstrating a commitment to ongoing learning and professional development. It also highlights the importance of providing accessible and relevant CPD opportunities for pharmacists of all ages to ensure they have the knowledge and skills to deliver high-quality patient care.

We observed that married pharmacists and those working longer hours were less likely to attend live on-site CPD activity. No specific information was found in the literature about the association between marital status, working hours, and attending live on-site CPD for pharmacists. One explanation for our findings could be that married pharmacists may have more family responsibilities that limit their time attending CPD activities. Moreover, pharmacists who work longer hours may have less time to attend CPD activities. This study highlighted the importance of considering individual factors, such as age and marital status, when designing CPD programs that are accessible and appealing to all pharmacists. However, we must note that these associations do not necessarily imply causation because of our study design. There is a need for further investigation into the factors that affect the participation of pharmacists in site CPD activities.

Female pharmacists attended online CPD less than males. This data suggests a gender difference in preferences for online-virtual CPD. In a university setting, a study found that female learners were significantly more likely to enroll in online courses than men [52]. Nonetheless, our results contradict the previous findings of Driesen et al., who reported that female pharmacists were more inclined towards attending lectures. In contrast, male pharmacists preferred distance learning more [53]. Further research is needed to understand the factors influencing gender differences in CPD preferences among pharmacists.

## Conclusion

This study is the first to explore pharmacists’ determinants of attending different CPD activities. Overall, most participants preferred live on-site attendance over virtual online attendance, with the interactive workshop being a popular choice as well. Printed materials were also reported to be more frequently used than video/Audio formats. The primary motivators for pharmacists to participate in CPD events were the topic and cost, while work responsibilities and family commitments were identified as the biggest obstacles. The cost of CPD activities and the distance required to travel to the site were also cited as significant barriers. Despite these obstacles, around one-third of the participants reported that attending CPD activities provided them with networking and socializing opportunities. Policymakers, regulators, pharmacy organizations, and pharmacists can use our findings to promote continuing education, improve programs, and implement evidence-based recommendations for safe patient care.

### Limitations and areas for future research

This study was conducted in the UAE, and our results may not be generalizable to other countries with diverse healthcare systems, cultural norms, and regulations. Therefore, future studies could aim to replicate our study in different countries to identify potential contextual or cultural factors influencing pharmacists’ participation. Furthermore, we did not investigate the impact of external factors on pharmacists' participation in CPD activities, such as regulatory requirements, financial incentives, and employer support. In addition, we used a cross-sectional design, which limits the ability to establish causality. Future research can examine external factors using a longitudinal study design to provide more robust evidence. Moreover, our data relied on self-reported questionnaires for participating in CPD activities over the previous 6 months, introducing potential response and recall biases that limit verifying the accuracy of the data. Future studies could use a mixed-methods approach that combines self-reported surveys and interviews to explore pharmacists’ views and preferences for CPD courses during a shorter time frame. In addition, the potential for a social desirability bias remains a limitation of the study, where participants provide responses socially acceptable or desirable rather than their true beliefs or behaviors [54]. While professional organizations undoubtedly offer valuable educational resources and networking opportunities through their CPD programs, it is essential to acknowledge that our investigation did not encompass an examination of the specific role played by professional organizations in supporting pharmacists through CPD programs. Future studies hold the potential for conducting more in-depth evaluations to assess the effectiveness of professional organizations in delivering CPD programs, scrutinize their impact on supporting pharmacists' professional development, and analyze the long-term outcomes resulting from pharmacist engagement with these organizations. Finally, it is important to acknowledge the limitation regarding the proportion of hospital pharmacists, which accounted for only 8.1% of the total sample. This limitation opens up opportunities for future research to further explore and expand upon the experiences and perceptions of hospital pharmacists in a more comprehensive manner. Addressing this limitation by conducting dedicated studies that focus exclusively on hospital pharmacists would contribute significantly to the broader understanding of pharmacy practice, patient care, and healthcare system dynamics.

## Data Availability

The questionnaire is available on the request.
